# Mental health issues impacting pharmacists during COVID-19

**DOI:** 10.1186/s40545-020-00252-0

**Published:** 2020-07-22

**Authors:** Ali Elbeddini, Cindy Xin Wen, Yasamin Tayefehchamani, Anthony To

**Affiliations:** 1 Winchester District Memorial Hospital, 566 Louise Street, Winchester, Ontario, KK0C2K0 Canada; 2 Leslie Dan Faculty of Pharmacy, University of Toronto, 144 College St, Toronto, M5S 3M2 Canada

**Keywords:** COVID-19, Mental health, Pharmacists

## Abstract

The coronavirus disease 2019 (COVID-19) impact on the mental health of healthcare workers is extremely detrimental. It is imperative that the psychological health of all healthcare workers be protected. However, an often overlooked member of the healthcare frontline is the pharmacist. Pharmacists provide many types of essential services during the pandemic, which often cannot be done from a remote location. Being frontline healthcare workers, pharmacists have experienced an increase in the number of patients seen, the amount of screening and triage being done, the amount of COVID-19 information being delivered, the number of medication shortages, and the amount of workplace harassment taking place. These activities increase the amount of stress, burden, and frustration felt by pharmacists have a negative impact on their mental health and well-being. This article seeks to address the specific implications of COVID-19 on the mental health of pharmacists.

## Background

COVID-19 is a severe infectious respiratory disease caused by a novel coronavirus (SARS-CoV-2) whose first case of infection occurred on December 31, 2019, in Wuhan, China [1]. The World Health Organization (WHO) has officially declared it a global pandemic on March 11, 2020 [2]. COVID-19 is transmitted person-to-person via droplets produced by coughing, sneezing, speaking, and through contact with contaminated surfaces [1]. A state of emergency was declared, leading to social distancing and closures of schools, and non-essential businesses across Canada [3]. These measures were implemented to control the physical spread of COVID-19 and ramifications on the population’s mental health.

The challenging conditions imposed causes increased stress, anxiety, depressive symptoms, and exacerbation of pre-existing mental illness [4–7]. Social isolation is strongly associated with poor mental health outcomes [8–10], especially in the context of COVID-19 [6, 11, 12]. If left unchecked, a mental health crisis could come out of the current COVID-19 pandemic. These mental health issues extend to healthcare workers (HCWs) who work on the front line to treat those who are infected [12]. HCWs worldwide report the negative psychological effects of COVID-19, such as stress, fear, anxiety, depression, burnout, and mental exhaustion [7, 12–19].

Pharmacists are one of the frontline HCWs working diligently to provide much-needed services during the pandemic [20–26]. Pharmacist-provided services have shown to improve patient outcomes and contribute to healthcare savings [26]. Community pharmacists provide COVID-19 screening and medication dispensing to maintain continuity of care, disseminate critical information regarding COVID-19, collaborate closely with other HCWs and government organizations, engage in home medication delivery, and remain the most accessible healthcare member that patients can interact with [25]. Hospital pharmacists directly support physicians, nurses, and other staff in managing life-saving medications for COVID-19 patients, participating in patient rounds, and engaging in infectious disease control [20, 25–27]. Unfortunately, pharmacists are often overlooked and are underrepresented regarding advocacy [25]. In this editorial, we will address pharmacist-specific mental health issues as well as methods to support pharmacists’ psychological well-being.

**Fig. 1:**
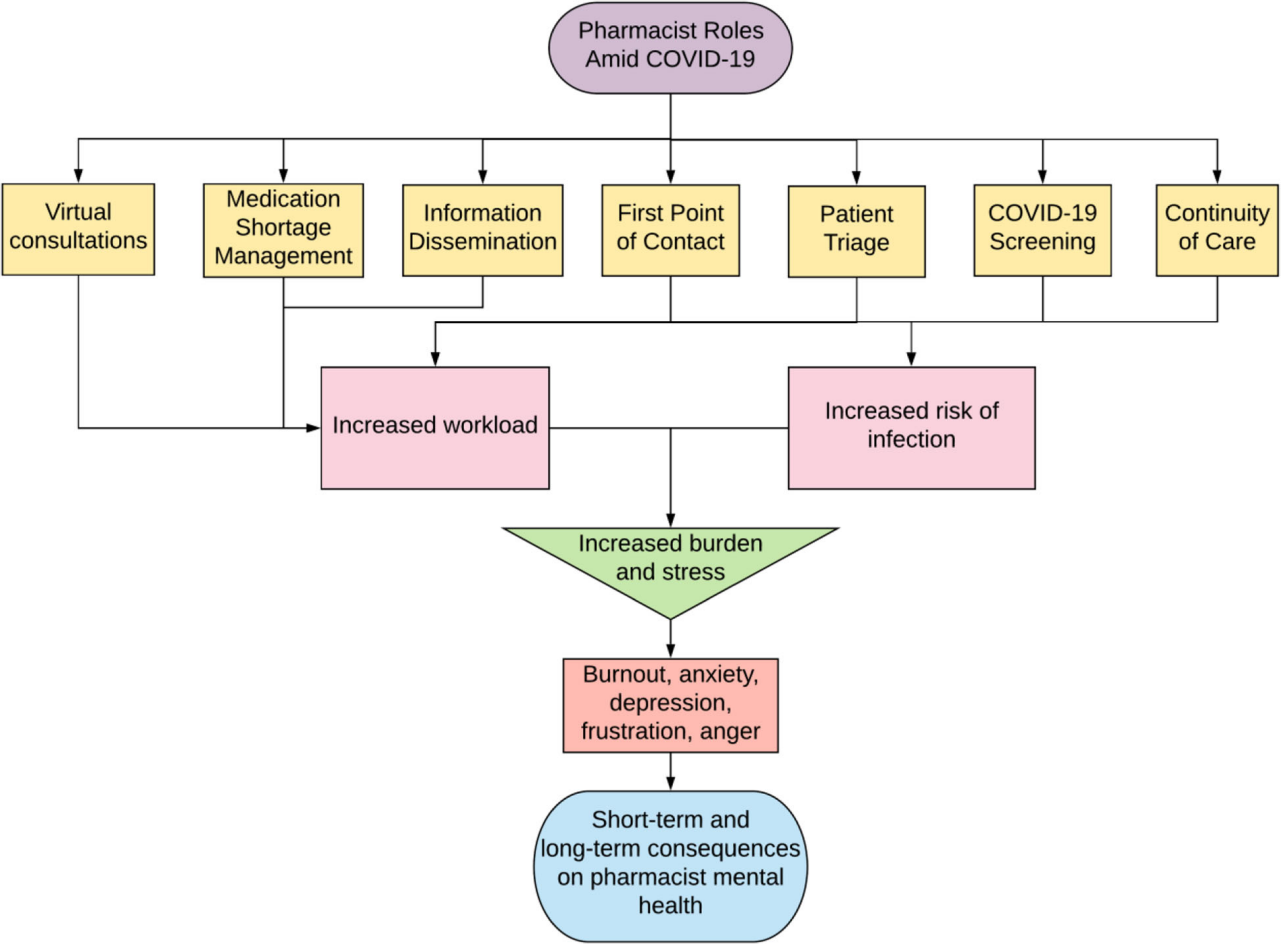
Pharmacist roles amid COVID-19 that can lead to decreased pharmacist mental health

Pharmacists experience issues common to all HCWs during COVID-19. Both community and hospital pharmacists experience a lack of personal protective equipment (PPE). Long work hours are required for treating the increased number of scared and frustrated patients, along with increased responsibility and pressure. Increased risk of infection resulting from work-related exposure leads HCWs to self-isolate, which can result in feelings of isolation and loneliness. However, due to the pharmacist’s unique role, they will also experience pharmacist-specific issues as summarized in Fig. 1.

## Increased burden on pharmacist roles leads to decreased pharmacist mental well-being

### First point of patient contact

As one of the most easily accessible HCWs, pharmacists are often the first point of contact for many patients. Pharmacists may experience an increase in the number of patients seen during the pandemic. A survey of nine major hospitals in the USA showed a significant decrease in the number of ST-segment elevation cardiac catheterization laboratory activations during the pandemic [28], indicating that there is a possibility that fewer patients are seeking hospital care. Understandably, many patients who have non-COVID-19-related illnesses may be hesitant to go directly to a hospital or other similar institutions for fear of contracting COVID-19 [29, 30]. Instead of going to a hospital, they may present themselves to a pharmacy to receive guidance from a pharmacist, which results in a decrease in the number of non-essential hospital visits and allows healthcare resources reallocation to treat COVID-19. However, this could lead to a strain on community pharmacists as they attempt to fill that clinical role. The resulting increased workload will add to the burden, leading to burnout among pharmacy staff, which is detrimental to individual well-being and compromising the quality of care provided [31].

### Patient triage

Pharmacists also engage in patient triage [32]. While there may exist some increased risk of contracting COVID-19 in clinics and hospitals, patients who need care should not be afraid to seek care. Pharmacists can provide over the phone, and in-person screening of COVID-19 symptoms as well as appropriately redirect patients to other healthcare facilities. The importance of continuity of care is amplified during a pandemic and demonstrates the importance of pharmacist triage. However, it is not always easy to triage and prioritize patients—difficult decisions often must be made. This also emphasizes the importance of adequate PPE, as screening exposes pharmacists to the risk of infection and contamination. Both the burden of appropriate triage and the absence of peace of mind against the disease contribute to the stress that pharmacists face.

### Information dissemination

As pharmacists provide essential information regarding COVID-19 to the public in an easy to access manner, they must stay up to date on the latest pandemic developments. Pharmacists help patients navigate fact from fiction as well as educate on proper hygiene and infection control. This is important as misconceptions about COVID-19 can exacerbate worry and other mental health concerns [5, 22]. However, pharmacists find themselves under constant bombardment of COVID-19 related information, which can be overwhelming and affect overall mental well-being [33]. The Canadian Pharmacist Association (CPhA) recommends that pharmacists limit the amount of time per day spent reviewing news, to only focus on the general trajectory, and to distract your mind with other tasks, among other suggestions [33]. Pharmacists can also use adverse drug reaction (ADR) and medical device incident (MDI) reporting to facilitate misinformation correction [34]. The demand for COVID-19 therapies drives the rampant adoption of potential medications, even when there is insufficient evidence for its efficacy and safety [34]. Thus, the vigilant reporting of ADRs is more critical than ever to combat misinformation.

### Managing medication shortages

Pharmacists are also managing medication shortages and limited resources during COVID-19 [35, 36]. Before the pandemic, medication shortages were already a global issue [20]. The spread of COVID-19 generated a spike in the number of critically ill patients and fear-based medication hoarding contributing to the pre-existing shortages [35, 36]. During the first week of May 2020, only 3% of pharmacies across Canada reported receiving their full medication order for every order placed [37]. Medication shortages disrupt care and pose safety concerns for patients. Two drugs that have been publicized as candidates for the treatment of COVID-19, hydroxychloroquine and chloroquine, have now become difficult to obtain for patients who need these agents to manage rheumatoid arthritis, systemic lupus erythematosus, and other autoimmune disorders [35, 36]. Canadian pharmacists are spending 24% of their shift dealing with medication shortages, placing a huge burden [37]. While most of medication shortage issues are out of the pharmacists’ control, there are some initiatives pharmacists have taken to tackle the problem. Pharmacists can find alternative sources, alternative therapies, and rationing existing drug supplies. One such proposed method is to sterilize metered-dose inhalers used once or twice in hospitals so that they can be reused [38]. In addition to technical difficulties in managing medication shortages, there are also ethical dilemmas when deciding to prioritize one patient over another, which can also be damaging to mental well-being. This results in something known as moral injury, described as “the psychological distress which results from actions, or lack of them, which violate someone’s moral or ethical code.” [39]

### Harassment from patients

An unfortunate circumstance that pharmacists have to face is harassment and abuse. In a national survey conducted by CPhA, 73% of pharmacists report an increase in harassment, verbal abuse, and other forms of abuse by patients ever since the pandemic began [40]. Anecdotal accounts also exist of healthcare workers experiencing stigma and abuse due to the public’s fear of contracting COVID-19 from someone who has high exposure to the virus [31, 41]. In such a perilous time, it is understandable that patients will feel frustrated, angry, and frightened. However, pharmacists do not deserve to be poorly treated. It is important to let them know that pharmacists are doing their best to support patient health throughout the pandemic and beyond. As a result of the power dynamics at play, it is ultimately up to pharmacists to be able to reassure patients and provide care, all while taking into account their mental health. Currently, guidelines regarding patient interaction during a pandemic are needed.

An additional burden Asian pharmacists face on top of pharmacist harassment is the rise of anti-Asian racism that has come about due to COVID-19. Verbal and even physical abuse has been reported to happen in various countries, such as the UK, France, and the USA, to those of Chinese descent [41, 42]. Asian frontline workers are being told that the virus “came from [their] kind” and going so far as to call SARS-CoV-2 the “Chinese virus” [41, 42]. These reactions are fueled by fear and misinformation, which speaks to the damage that this kind of misinformation can cause if not corrected. Such racism should not be tolerated, seeing as it has such profound adverse effects on pharmacists and individuals’ well-being, community cohesiveness, and impeding the fight against COVID-19. Additional research is needed to address the mental health and long term consequences of anti-Asian sentiments and to develop initiatives and policies that seek to reduce such behaviors.

## Significance

Mental health care for HCWs is more critical now than ever. According to CPhA, pharmacists’ mental health was among the top four pharmacists’ greatest concerns during COVID-19, along with personal and staff safety, drug shortages, and workload/staffing shortages [40]. Poor HCW mental health may lead to decreased quality of care, attention paid, and decision-making ability [13], but pharmacist-specific data is lacking. One study looked at the job satisfaction of hospital pharmacists in Ethiopia [43], which can be considered another indicator of the pharmacists’ well-being. Pharmacists with low job satisfaction often have less productivity and lower overall quality of life [43]. On the contrary, high job satisfaction positively impacts performance, employee relationships, mental health, and life satisfaction [43]. During such dire times, pharmacists are being relied upon more and more to provide much-needed services during the pandemic. Pharmacists, like other HCWs, know how important their role is and are resilient in providing care even at the expense of their own mental and physical well-being [24, 44]. It is well known that mental illness is associated with lower life expectancy and poorer health outcomes than the general population, increasing the risk of infection with COVID-19 [45]. It is uncertain what exactly the long-term sequelae may be, but there is evidence that such sequelae exist. It was found that 3 years after the SARS outbreak in 2003, high-risk HCWs remained highly stressed, which was associated with higher levels of depression, anxiety, and general psychological distress [46–48]. The stress generated by COVID-19 for HCWs is akin to that of a natural disaster or international mass conflict [5]. While it might make sense during the beginning of the outbreak to prioritize physical health or psychological, it should not stay this way. COVID-19 is as much of a somatic battle as it is a mental battle. If unaddressed, poor mental health can have more serious consequences down the line and may lead to a shortage of pharmacists and other HCWs after the pandemic is over [5].

## Future directions

While they do exist, the number of resources offered to manage mental health is scant. CPhA provides infographics and links to province-specific pharmacy mental health resources, and nationwide and American pharmacist resources [33, 49]. Multidisciplinary mental health services must also be explicitly provided for pharmacists and other HCWs as they experience more extreme psychologic symptoms [19]. More awareness should be generated for these resources and the importance of pharmacists’ mental health during COVID-19.

Healthcare delivery is progressing in the direction of telemedicine and virtual methods of delivery. Mental health is a very good candidate for electronic delivery [50, 51]. This can be extended to providing mental health care to pharmacists and other HCWs. Blake et al. assessed a digital learning package that consisted of evidence-based guidance, actions to take, and self-care strategies relating to maintaining mental well-being for HCWs in the UK [31]. They found that it was well-received with high user satisfaction [31]. The material was better received if it addressed specific issues that were relevant to healthcare work environments and the type of work healthcare workers engaged in [31]. This further proves the importance of having more pharmacy-specific mental health resources. Besides focusing on the individual, fostering a culture of resilience and support within an organization strongly affects worker stress and protects mental health [31, 39, 52]. In addition to providing care, pharmacists must also have tools to assess mental health in the context of COVID-19 to identify individuals at risk quickly and not to over- or under-diagnose [53]. Finally, it would be good to learn from previous pandemics and other countries regarding their response to HCW mental health. Being the epicenter of COVID-19, China had some rapid response to improving the mental health of their HCWs [7]. The Second Xiangya Hospital in China’s Hubei province developed a detailed psychological intervention plan, a psychological assistance hotline team and psychological interventions to improve HCW mental health [54]. The National Health Commission of China integrated psychological crisis intervention into general measures for disease prevention, issued the “Principles for Emergency Psychological Crisis Intervention for COVID-19 Pneumonia Epidemic,” as well as a plethora of mental health guidelines made by various healthcare organizations [55].

Overall, the pharmaceutical system itself could be strengthened, which could go beyond just mitigating drug shortages and help pharmacist workflow and well-being. Hafner et al. report three issues that stand in the way of low- and middle-income countries being able to strengthen their pharmaceutical systems [56], which also applies to Canada as its pharmaceutical systems become strained due to COVID-19 and issues become apparent. Firstly, the process of strengthening a pharmaceutical system is lengthy and resource intensive [56], which may not be feasibly done in time during COVID-19. Secondly, strengthening pharmaceutical systems requires the engagement of government bodies and multiple stakeholders as well as policy and legislation reform [56], which Canada is currently engaging in for managing the immediate COVID-19 threat. Finally, the interventions that work to strengthen pharmaceutical systems are not precisely known, which is an area of further research [56].

## Summary

The mental health of HCWs during the COVID-19 pandemic needs to be addressed. Pharmacists are frontline HCWs and belong in this category as they provide necessary services amid the pandemic. As one of the most accessible HCWs, they face many stressors that need to be specifically targeted to address pharmacists mental health issues effectively. All in all, more research and resources need to be developed to provide solutions for the declining psychological well-being of pharmacists and HCWs in general amid COVID-19.

## Data Availability

Data sharing does not apply to this article as no datasets were generated or analyzed during the current study.
